# Sodium Copper Chlorophyllin Inhibits Porcine Reproductive and Respiratory Syndrome Virus Infection Through Multiple Antiviral Mechanisms

**DOI:** 10.1155/tbed/6975628

**Published:** 2026-06-02

**Authors:** Xinrong Wang, Juan Zhang, Li Rui, Junhai Zhu, Nan Yan, Meiyu Jia, Longxiang Zhang, Lizhi Fu, Yue Wang

**Affiliations:** ^1^ College of Veterinary Medicine, Southwest University, Chongqing, 400715, China, swu.edu.cn; ^2^ Veterinary Medicine and Pharmaceuticals Research Institute, Chongqing Academy of Animal Sciences, Chongqing, 402460, China; ^3^ National Center of Technology Innovation for Pigs, Chongqing, 402460, China; ^4^ Rongchang Field Scientific Observation and Research Station for Animal Diseases, Ministry of Agriculture and Rural Affairs, Chongqing, 402460, China, agri.gov.cn

**Keywords:** antiviral drugs, PRRSV, sodium copper chlorophyllin

## Abstract

Porcine reproductive and respiratory syndrome virus (PRRSV) remains a major threat to the global swine industry due to its extensive genetic diversity and limited vaccine cross‐protection. Antiviral strategies that target both viral infection and host pathological responses are urgently needed. Here, we evaluated the antiviral efficacy and underlying mechanisms of sodium copper chlorophyllin (SCC) against PRRSV infection. In vitro analyses demonstrated that SCC exhibited potent and dose‐dependent inhibitory activity against both classical and highly pathogenic PRRSV‐2 strains in Marc‐145 cells and immortalized porcine alveolar macrophages (iPAMs). Cotreatment produced the strongest antiviral effect, indicating that SCC primarily targets early stages of viral infection. Mechanistically, SCC directly disrupted viral particle integrity, resulting in viral RNA release and loss of infectivity. In addition, SCC significantly inhibited viral adsorption, internalization, and intracellular genome replication, while exerting no detectable effect on progeny virion release. SCC also reduced the expression of the key PRRSV entry receptor CD163 in porcine macrophages, thereby impairing virus‐host interactions. Furthermore, SCC markedly alleviated PRRSV‐induced oxidative stress and inflammatory responses by reducing intracellular reactive oxygen species (ROS) and enhancing antioxidant gene expression (heme oxygenase‐1 [*HO-1*], NAD(P)H quinone oxidoreductase 1 [*NQO1*], and glutamate–cysteine ligase modifier subunit [*GCLM*]), while suppressing proinflammatory cytokines (TNF‐α, IL‐6, and IL‐8). Network pharmacology analysis supported the involvement of oxidative stress‐ and metabolism‐related pathways. Importantly, in a PRRSV challenge model, oral administration of SCC significantly reduced viral loads, alleviated fever, and attenuated lung pathology. Collectively, SCC displays broad antiviral activity through direct virucidal effects, inhibition of early viral entry, and modulation of host responses, highlighting its potential as a therapeutic candidate for PRRSV control.

## 1. Introduction

Porcine reproductive and respiratory syndrome virus (PRRSV) remains one of the most economically devastating pathogens in the global swine industry, causing reproductive failure in sows and severe respiratory disease in growing pigs [1]. Recent analyses estimate annual economic losses ranging from hundreds of millions to over a billion US dollars in major producing regions [2–4]. Despite widespread use of modified‐live and inactivated vaccines, effective control of PRRSV remains challenging due to its remarkable genetic diversity, complex immune evasion capabilities, and incomplete cross‐protection among heterologous strains [5–7]. Moreover, there are currently no approved antiviral drugs available for clinical use in pigs, leaving a critical gap in therapeutic options after infection is established. Increasing evidence indicates that PRRSV pathogenesis is not only driven by viral replication but also by dysregulated host responses [8–10], including excessive inflammatory activation [11, 12] and oxidative stress in infected macrophages [13, 14], which contribute to viral persistence and tissue damage. Therefore, antiviral strategies that both suppress viral replication and modulate host pathological responses are urgently needed to complement existing vaccination programs.

Sodium copper chlorophyllin (SCC), a water‐soluble copper complex of chlorophyll, has recently emerged as a promising broad‐spectrum antimicrobial agent [15]. Previous studies have demonstrated its antiviral ability against Enterovirus A71 (EV‐A71) through the inhibition of viral attachment and entry [16]. Mechanistically, metal‐chlorophyllin complexes are reported to exert biological effects through redox regulation, metal‐ion‐mediated disruption of viral components, and modulation of reactive oxygen species (ROS) generation [15]. In addition, SCC exhibits antibacterial and antibiofilm activities and has shown a favorable safety profile in preclinical studies, supporting its translational potential [17–19]. However, its antiviral efficacy and underlying mechanisms against economically important animal viruses, such as PRRSV, remain largely unexplored.

Given the critical role of oxidative stress and inflammatory dysregulation in PRRSV pathogenesis [20], we hypothesized that SCC may function as a multifunctional antiviral agent by targeting both viral infection and host pathological responses. In this study, we investigated the antiviral activity and mechanisms of SCC against PRRSV in vitro, focusing on its effects on viral attachment, entry, and replication, as well as its regulation of host oxidative stress and inflammatory signaling. Furthermore, the antiviral findings were validated in vivo using a PRRSV challenge model in piglets to evaluate the therapeutic potential of SCC in reducing viral load, alleviating clinical symptoms, and mitigating pathological lesions. Collectively, these findings support SCC as a promising host‐directed antiviral candidate for PRRSV control in the global swine industry.

## 2. Materials and Methods

### 2.1. Cells and Viruses

Marc‐145 cells were maintained in Dulbecco’s modified Eagle medium (DMEM) supplemented with 10% fetal bovine serum (FBS) and 1% penicillin‐streptomycin at 37°C in a humidified 5% CO_2_ incubator. Primary porcine alveolar macrophages (PAMs) and immortalized PAMs (iPAMs) were maintained in RPMI 1640 medium supplemented as described above. PRRSV strains CH1R (GenBank: EU807840.1, classical), BJ‐4 (GenBank: AF331831, classical), HuN4 (GenBank: EF635006, highly pathogenic), Hn07‐1 (GenBank: KX766378.1), and JXA1 (GenBank: EF112445.1, highly pathogenic) were propagated in Marc‐145 cells. Virus titers were determined by the 50% tissue culture infectious dose (TCID_50_) assay. All cells and viruses were maintained and stored in our laboratory.

### 2.2. Inhibitory Effects of SCC on PRRSV Infection

Marc‐145 and iPAM cells were seeded into 12‐well plates and cultured to 80%–90% confluence before PRRSV infection at a multiplicity of infection (MOI) of 0.1. Three treatment strategies were employed to evaluate the inhibitory effects of SCC on viral infection. In the pretreatment group, cells were exposed to SCC for 1 h prior to PRRSV infection, after which the unbound virus was removed by washing, and cells were cultured for an additional 24 h. In the cotreatment group, SCC and PRRSV were added simultaneously and incubated for 1 h, followed by washing and incubation for an additional 24 h. In the post‐treatment group, cells were first infected with PRRSV for 1 h, washed to remove unabsorbed virus, and then incubated with SCC‐containing medium for 24 h. Viral infection was evaluated by quantifying PRRSV ORF7 mRNA levels using RT‐qPCR, assessing N protein expression by Western blot, and performing immunofluorescence staining with anti‐PRRSV GP5 antibody to visualize viral proteins. The primers used for qPCR are shown in Table 1, and the annealing temperature for all reactions was set at 60°C.

**Table 1:** The primers used in this study.

Gene name	Sense primers (5′ to 3′)	Antisense primers (5′ to 3′)	Amplicon (bp)
PRRSV‐ORF7	agatcatcgcccaacaaaac	gacacaattgccgctcacta	144
Porcine‐β‐actin	cttcctgggcatggagtcc	ggcgcgatgatcttgatcttc	201
Monkey‐β‐actin	atcgtgcgtgacattaag	attgccaatggtgatgac	135
CD163	gggcaagtggcctctgtaat	ccccaggagccctcatgata	200
IL‐6	aaaaggtgggtgtgtcctcc	tggcattgcatccctgagtt	91
IL‐8	tggaccacactgcgtcaata	tccacaaccctagacaccca	99
TNF‐α	cagctggagaagggtgatcg	ctcacagggcaatgatccca	103
PPARG	aacatttcacaagaggtgacca	cagctctcgggaatgggatg	190
EGFR	aaagagtaccacgcggaagg	ttctccaggacggtcgagat	188
IDH1	tgcccatgggactgtaacac	aagccagcctcaatggtctc	192
INS	cttcttctacacgcccaaggc	ctggtagagggaacagatgct	172
HO‐1	cagcaacaaagcgcaagact	acgtaaggacccatcggaga	101
NQO1	agggatccatggggacatga	ccaggcgtttcttccatcct	161
GCLM	atcttgcctcctgctgtgtg	ctcgtgcgcttgaatgtcag	158

### 2.3. Virucidal Activity of SCC

To assess direct virucidal activity, PRRSV (MOI = 1) was incubated with SCC at concentrations of 0, 40, and 100 μM at 37°C for 1 h. The mixtures were then added to Marc‐145 cell monolayers for 1 h, after which the unbound virus was removed by washing, and cells were cultured in DMEM containing 2% FBS for 24 h. Viral RNA levels were measured by RT‐qPCR to determine the inhibitory effect of SCC. For absolute quantification of viral RNA, PRRSV was incubated with SCC over a wider concentration range (0–200 μM) at 37°C for 1 h. Viral titers were determined by the TCID_50_ assay, and viral RNA genome copy numbers were directly quantified by RT‐qPCR without prior RNA purification or virus inactivation, allowing the assessment of SCC‐induced disruption of viral particles.

### 2.4. Inhibition of PRRSV Replication Cycle

To examine SCC the effects on specific stages of the viral life cycle, adsorption, entry, replication, and release assays were performed. For adsorption inhibition, Marc‐145 cells were pretreated with SCC (0, 20, 40, and 200 μM) at 37°C for 1 h, chilled at 4°C for 20 min, and then infected with PRRSV (MOI = 1) at 4°C for 1 h. Unbound virus was removed by washing, and viral attachment was assessed by RT‐qPCR of ORF7 and by confocal microscopy using an anti‐GP5 antibody. To evaluate entry inhibition, cells were first incubated with PRRSV at 4°C for 1 h, washed to remove the unbound virus, and then incubated at 37°C with SCC‐containing medium for 2 h. Following removal of the non‐internalized virus, cells were lysed for RT‐qPCR analysis of viral RNA. For replication inhibition, Marc‐145 cells were infected with PRRSV (MOI = 1) for 1 h, washed, and cultured for 2 h in a maintenance medium before adding SCC (200 μM). Cell lysates were collected at 6, 8, and 10 h postinfection (hpi), and viral RNA levels were quantified. To assess viral release, cells were infected with PRRSV (MOI = 1) for 1 h, washed to remove the unabsorbed virus, and cultured until 7 hpi, when the SCC‐containing medium was added. Supernatants were collected at multiple time points (8–24 hpi), and viral RNA was quantified to determine the effect of SCC on progeny virus release.

### 2.5. Receptor Expression Analysis

PAMs and iPAMs were treated with SCC (100 or 200 μM) for 1 h. The mRNA and protein expression levels of the key PRRSV receptor, CD163, were subsequently analyzed by RT‐qPCR and Western blot, respectively, with β‐actin as an internal control. To determine the degradation pathway involved in SCC‐mediated CD163 downregulation, iPAMs were pretreated with the proteasome inhibitor MG132 (1 μM) or the lysosome inhibitor bafilomycin A1 (Baf‐A1, 1 μM) for 6 h, followed by SCC treatment. CD163 protein levels were then assessed by Western blot to assess the involvement of proteasomal or lysosomal degradation pathways.

### 2.6. Inflammatory Cytokine Assay

Marc‐145 cells were infected with PRRSV (MOI = 1) and treated with SCC under pre‐, co‐, or post‐treatment conditions. Total RNA was extracted at 12, 24, 36, and 48 hpi, and the mRNA levels of TNF‐α, IL‐6, and IL‐8 were quantified by RT‐qPCR using β‐actin as the reference gene to evaluate the modulatory effects of SCC on PRRSV‐induced proinflammatory responses.

### 2.7. Oxidative Stress Assay

Intracellular ROS levels were measured in Marc‐145 cells infected with PRRSV (MOI = 1) and treated with SCC (200 μM) using the DCFH‐DA (2′,7′‐dichlorodihydrofluorescein diacetate) fluorescent probe at 24 hpi. The fluorescence intensity was quantified using a microplate reader. Heme oxygenase‐1 (*HO-1*), NAD(P)H quinone oxidoreductase 1 (*NQO1*), and glutamate–cysteine ligase modifier subunit (*GCLM*) mRNA levels were measured by RT‐qPCR to assess the SCC‐mediated activation of antioxidant defenses. Intracellular levels of GSH (reduced glutathione, Beyotime, China) were detected with a commercial assay kit according to the manufacturer’s instructions.

### 2.8. Network Pharmacology Analysis

Potential PRRSV‐related target genes were retrieved from the CTD and GeneCards databases and intersected with SCC‐associated targets to identify candidate genes for SCC‐mediated antiviral activity. Protein–protein interaction (PPI) networks were constructed using STRING and Cytoscape (3.10.2), and hub genes were identified based on the network topology. Gene Ontology (GO) and KEGG pathway enrichment analyses were performed to characterize the biological processes, cellular components, molecular functions, and pathways associated with SCC targets. A “SCC‐targets‐pathways” interaction network was constructed, and key nodes were identified to explore the potential molecular mechanisms of SCC against PRRSV.

### 2.9. Animal Experiment

Using a computer‐generated random number table, 12 4‐week‐old PRRSV‐free piglets (~5 kg) were randomly assigned to three groups (*n* = 3 per group): control (no virus or treatment), HuN4 (virus challenge only), and the HuN4 + SCC group (HuN4 challenge + SCC). Piglets in the HuN4 and HuN4 + SCC groups were intramuscularly challenged with 5 × 10^4^ TCID_50_ of the PRRSV HuN4 strain, while the control group received sterile PBS. At 1 dpi, piglets in the HuN4 + SCC group were orally administered SCC (50 mg/kg body weight) once daily for three consecutive days (1–3 dpi). This treatment plan was based on previous research showing that early intervention most effectively reduces viral load and disease severity, particularly given that PRRSV replication peaks during the early stage of infection (3–7 dpi) [7]. Body weights were recorded at 0, 2, 4, 6, 8, 10, 12, and 14 dpi. Rectal temperature and clinical signs were monitored daily. Serum samples were collected at 0, 1, 3, 5, 7, 10, and 14 dpi for viral RNA quantification via RT‐qPCR. Lung tissues collected at 14 dpi were analyzed for viral RNA levels and histopathological changes using H&E staining. Pulmonary health was assessed based on gross lesions, using a subjective scoring system to estimate the extent of pneumonia using a recognized method [21]. In parallel, microscopic lung lesions were scored on a 0–4 scale to indicate the severity of interstitial pneumonia [22].

### 2.10. Statistical Analysis

All experiments were performed with at least three independent biological replicates. Data are presented as the mean ± standard deviation (SD) from at least three independent experiments. Comparisons between two groups were conducted using an unpaired, two‐tailed Student’s *t*‐test, while comparisons among multiple groups were analyzed using one‐way analysis of variance (ANOVA). Statistical analyses were performed using GraphPad Prism (version 8.0). Statistical significance was defined as follows: ns, *p* > 0.05;  ^∗^, *p* < 0.05;  ^∗∗^, *p* < 0.01;  ^∗∗∗^, *p* < 0.001;  ^∗∗∗∗^, *p* < 0.0001 versus control group.

## 3. Results

### 3.1. SCC Exhibits Potent Antiviral Activity Against PRRSV In Vitro

**Figure 1:**
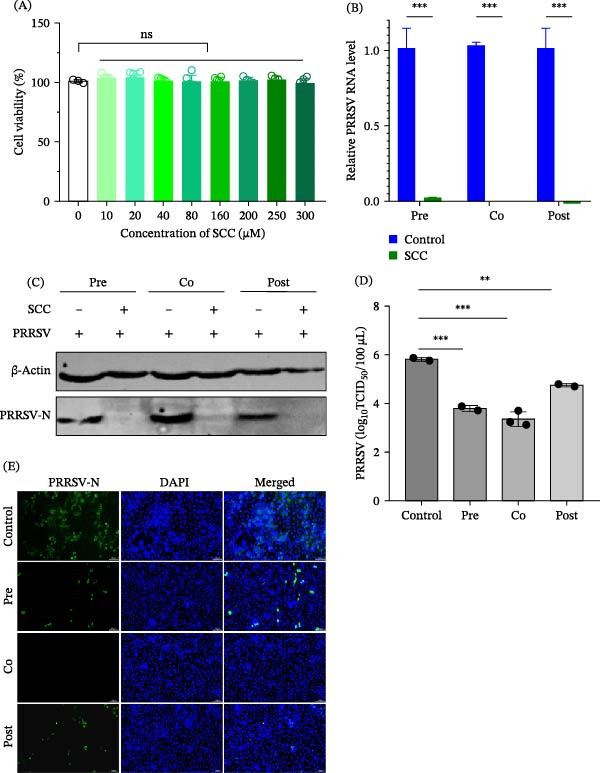
SCC exhibits potent antiviral activity against PRRSV in Marc‐145 cells. (A) Cytotoxicity of SCC in Marc‐145 cells determined by a CCK‐8 assay after 24 h of treatment. (B) Quantification of PRRSV RNA levels by RT‐qPCR in pre‐, co‐, and post‐treatment groups. (C) Western blot analysis of PRRSV N protein expression under different treatment conditions. (D) Viral titers in supernatants from the same treatment groups measured by TCID_50_ assay. (E) Immunofluorescence staining of PRRSV N protein (green) showing markedly reduced fluorescence in SCC‐treated cells. Nuclei were counterstained with DAPI (blue). Data represent the mean ± SD from three independent experiments. ns, *p* > 0.05;  ^∗∗^, *p* < 0.01;  ^∗∗∗^, *p* < 0.001 versus virus control.

Cytotoxicity testing using a CCK‐8 assay showed that SCC exhibited no detectable toxicity in Marc‐145 cells at concentrations up to 300 µM (Figure 1A). To assess its antiviral efficacy against PRRSV, viral RNA levels were quantified by RT‐qPCR. Compared with the untreated control, SCC treatment significantly reduced viral RNA expression in the pre‐, co‐, and post‐treatment groups (Figure 1B). Western blot analysis further confirmed this effect, showing decreased viral N protein expression under all treatment conditions (Figure 1C). Consistent with these findings, viral titers were markedly reduced in all three treatment strategies, with the cotreatment group exhibiting the strongest antiviral effect (Figure 1D). IFA analysis supported these results, revealing a markedly reduced fluorescence intensity of the PRRSV N protein in SCC‐treated cells (Figure 1E).

**Figure 2:**
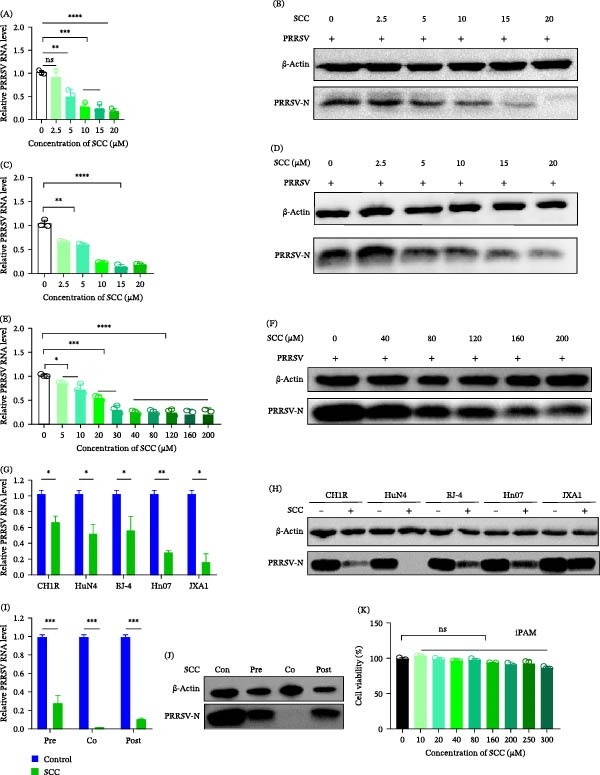
SCC inhibits PRRSV replication in a dose‐dependent manner and exhibits broad antiviral efficacy across PRRSV strains and cell types. (A–F) Marc‐145 cells were treated with increasing concentrations of SCC and then infected with PRRSV. Viral RNA and N protein expression were quantified by RT‐qPCR and Western blot, respectively. (G,H) Antiviral effects of SCC against classical (CH1R, BJ‐4) and highly pathogenic (HuN4, Hn07, and JXA1) PRRSV strains in Marc‐145 cells. (I,J) Validation of SCC antiviral activity in iPAMs by RT‐qPCR and Western blot analysis. (K) Cytotoxicity of SCC in iPAMs determined using a CCK‐8 assay. Data are presented as mean ± SD (*n* = 3). ns, *p* > 0.05;  ^∗^, *p* < 0.05;  ^∗∗^, *p* < 0.01;  ^∗∗∗^, *p* < 0.001;  ^∗∗∗∗^, *p* < 0.0001 versus control.

Within the noncytotoxic range, SCC inhibited PRRSV replication in a dose‐dependent manner, with cotreatment yielding the greatest reduction in viral RNA and N protein levels (Figure 2A–F). Moreover, SCC significantly suppressed viral RNA and N protein expression across multiple PRRSV strains, including the classical strains CH1R and BJ‐4 and highly pathogenic strains HuN4, Hn07−1, and JXA1 (Figure 2G,H).

To verify these effects in porcine target cells, its activity was further examined in immortalized porcine alveolar macrophages (iPAMs). RT‐qPCR and Western blot analyses revealed significant reductions in viral RNA and N protein levels in the pre‐, co‐, and post‐treatment groups, with cotreatment again showing the most pronounced inhibition (Figure 2I,J). Cytotoxicity testing confirmed that SCC was nontoxic to iPAMs at concentrations up to 300 µM (Figure 2K). Collectively, these findings demonstrate that SCC potently suppresses PRRSV replication, with cotreatment showing the most consistent antiviral protection.

### 3.2. SCC Exerts Direct Virucidal Activity Against PRRSV

**Figure 3:**
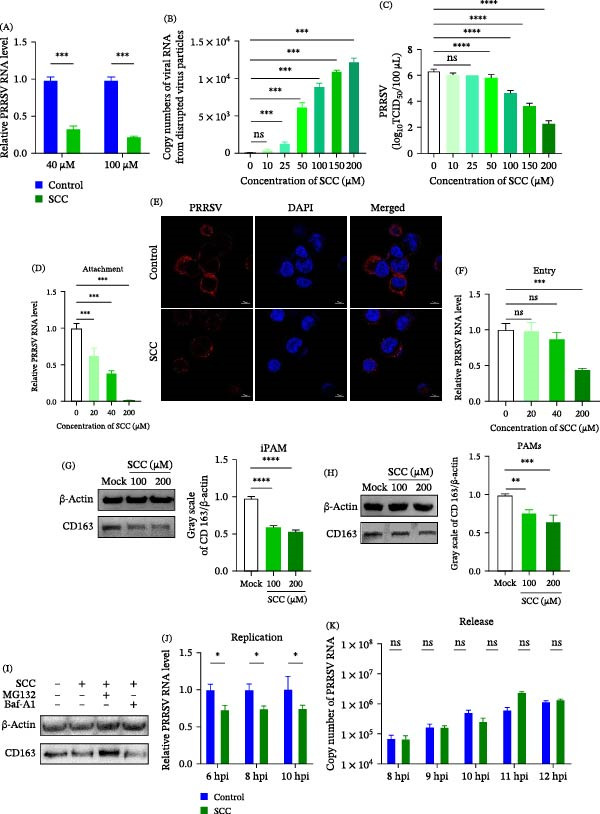
SCC interferes with multiple stages of the PRRSV life cycle. (A) PRRSV particles were incubated with various concentrations of SCC (0–200 μM) at 37°C for 1 h prior to infection of Marc‐145 cells. Viral RNA levels were measured by RT‐qPCR at 24 hpi. (B) Quantification of free viral RNA released after SCC treatment, measured by absolute qPCR without prior viral inactivation or RNA extraction. (C) Viral titers after incubation with SCC were measured by a TCID_50_ assay. (D) Adsorption inhibition assay. PRRSV binding to Marc‐145 cells pretreated with SCC (0, 20, 40, and 200 μM) at 4°C for 1 h was quantified by RT‐qPCR. (E) Representative confocal microscopy images showing cell surface‐attached PRRSV virions detected by anti‐GP5 monoclonal antibody staining following SCC treatment. (F) After viral attachment at 4°C, cells were incubated with SCC at 37°C for 2 h, and internalized viral RNA was quantified by RT‐qPCR. (G,H) Western blot analysis of CD163 protein in iPAMs (G) and primary PAMs (H) following SCC treatment. (I) Western blot analysis of the effect of MG132 or Baf‐A SCC‐induced CD163 protein degradation in iPAMs. (J) Viral replication assay. SCC was added after viral entry, and intracellular viral RNA levels were quantified by RT‐qPCR at 6, 8, and 10 hpi. (K) Viral release assay. SCC was added at 7 hpi, and culture supernatants were collected at 8–12 h for viral RNA quantification. Data are represented as mean ± SD (*n* = 3). ns, *p* > 0.05;  ^∗^, *p* < 0.05;  ^∗∗∗^, *p* < 0.001;  ^∗∗∗∗^, *p* < 0.0001 versus control.

To determine whether SCC exerts a direct virucidal effect, PRRSV was incubated with increasing concentrations of SCC at 37°C for 1 h prior to the infection of Marc‐145 cells. RT‐qPCR analysis revealed a dose‐dependent reduction in viral RNA at 24 hpi compared with the untreated control (Figure 3A), indicating that SCC directly impairs PRRSV infectivity. To further assess whether SCC disrupts viral particle integrity, PRRSV was incubated with SCC (0–200 μM) at 37°C for 1 h. Viral RNA copy numbers were then quantified directly by absolute qPCR without prior RNA extraction, while viral titers were simultaneously determined by a TCID_50_ assay. Notably, the amount of free viral RNA increased significantly with increasing SCC concentrations (Figure 3B), reaching approximately a 30‐fold increase compared with the untreated controls at 200 μM. Consistently, viral titers in the 200 μM SCC treatment group decreased by approximately 10,000‐fold compared with the untreated control (Figure 3C). These results suggest that SCC directly impairs the integrity of the PRRSV envelope, leading to viral RNA release and a consequent loss of viral infectivity.

### 3.3. SCC Inhibits Multiple Stages of the PRRSV Life Cycle

To further explore the SCC’s antiviral mechanism, we investigated its effects on distinct stages of the PRRSV life cycle, including viral adsorption, internalization, replication, and release. During the adsorption stage, RT‐qPCR analysis revealed a dose‐dependent reduction in virions attached to Marc‐145 cells following SCC treatment (Figure 3D). Consistently, immunofluorescence staining using an anti‐GP5 monoclonal antibody demonstrated markedly fewer virions attached to SCC‐treated cells compared with those of the control (Figure 3E), confirming that SCC effectively inhibits viral adsorption.

The effect of SCC on viral internalization was subsequently assessed. RT‐qPCR analysis showed that lower SCC concentrations had minimal effects, whereas higher concentrations significantly inhibited PRRSV internalization (Figure 3F). To elucidate the underlying mechanism, we examined the impact of SCC on CD163, a key cellular receptor required for PRRSV entry [23, 24]. iPAMs were pretreated with SCC for 1 h, followed by the analysis of CD163 expression. RT‐qPCR revealed no significant changes in CD163 mRNA levels (data not shown). In contrast, a pronounced reduction in CD163 protein expression was observed upon SCC treatment (Figure 3G). Similar results were observed in primary PAMs (Figure 3H), suggesting that SCC selectively downregulates CD163 at the protein level.

To verify the mechanism underlying SCC‐mediated CD163 downregulation, we used the proteasome inhibitor MG132 or the lysosome inhibitor Baf‐A1 to distinguish between proteasomal and lysosomal degradation pathways. As shown in Figure 3I, the SCC‐induced reduction of CD163 protein levels was substantially restored by MG132 cotreatment, whereas Baf‐A1 failed to prevent CD163 degradation. These data demonstrate that SCC promotes CD163 degradation specifically through the ubiquitin‐proteasome system rather than lysosomal pathways. Collectively, these findings suggest that SCC disrupts virus‐host interactions essential for PRRSV adsorption and membrane fusion during the early stage of infection by targeting CD163 for proteasomal degradation.

To determine whether SCC also affects viral genome replication, intracellular viral RNA levels were quantified at 6, 8, and 10 hpi following SCC treatment. SCC treatment markedly reduced intracellular viral RNA abundance compared with that of the control group (Figure 3J), indicating effective suppression of viral replication.

Finally, the potential impact of SCC on progeny virion release was evaluated. SCC was added at 7 hpi, and viral supernatants were collected at 8–12 hpi for RT‐qPCR analysis. No significant differences in viral RNA levels were observed between SCC‐treated and control samples (Figure 3K), indicating that SCC does not interfere with viral release. Collectively, these data demonstrate that SCC interferes with multiple early stages of the PRRSV life cycle, including viral adsorption, internalization, and genome replication, while having no detectable effect on progeny virion release.

### 3.4. SCC Mitigates Oxidative Stress Inflammatory Responses Induced by PRRSV Infection

**Figure 4:**
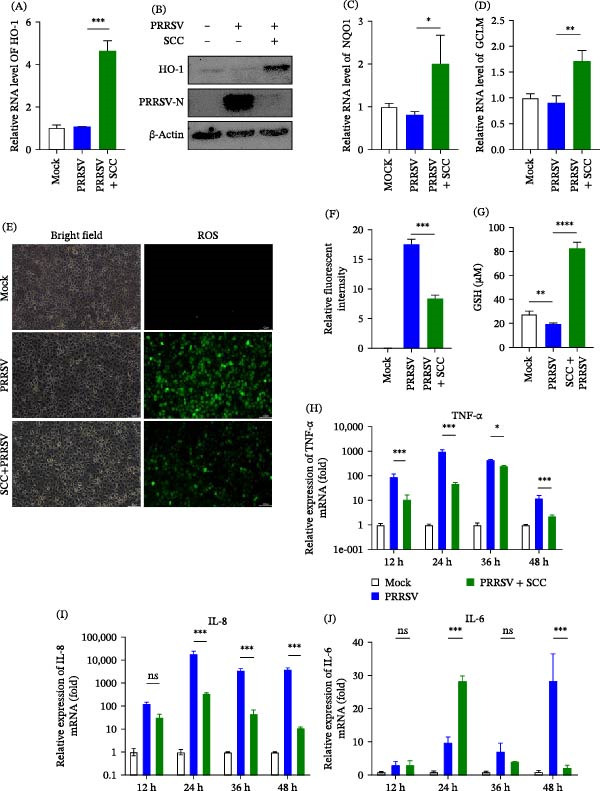
SCC mitigates oxidative stress and inflammatory cytokine expression induced by PRRSV infection. (A) RT‐qPCR analysis of HO‐1 mRNA expression in SCC‐treated, PRRSV‐infected Marc‐145 cells compared with controls. (B) Western blot analysis of HO‐1 protein expression in SCC‐treated, PRRSV‐infected Marc‐145 cells compared with controls. (C,D) RT‐qPCR analysis of NQO1 and GCLM mRNA expression in SCC‐treated, PRRSV‐infected Marc‐145 cells compared with controls. (E,F) Fluorescence microscopy of intracellular ROS using DCFH‐DA staining. Scale bar = 20 μm. (G) Determination of intracellular GSH concentration using a commercial kit in SCC‐treated, PRRSV‐infected Marc‐145 cells compared with controls. (H–J) RT‐qPCR analysis of TNF‐α (H), IL‐8 (I), and IL‐6 (J) mRNA levels at 12, 24, and 36 hpi in the presence or absence of SCC. Data are presented as mean ± SD (*n* = 3).  ^∗^, *p* < 0.05;  ^∗∗^, *p* < 0.01;  ^∗∗∗^, *p* < 0.001;  ^∗∗∗∗^, *p* < 0.0001 versus virus control.

To determine whether SCC directly modulates oxidative stress during PRRSV infection, the expression of key antioxidant enzymes, including HO‐1, NQO1, and GCLM, was evaluated. At 24 hpi, SCC treatment significantly upregulated the mRNA expression of HO‐1, NQO1, and GCLM, as well as the protein level of HO‐1, compared with PRRSV alone (Figure 4A–D), suggesting activation of the host antioxidant defense system. Consistently, fluorescence microscopy revealed that intracellular ROS accumulation was increased in PRRSV‐infected cells, whereas SCC treatment markedly reduced ROS levels (Figure 4D–E). SCC treatment also significantly increased the intracellular concentration of GSH (Figure 4G). These results indicate that SCC alleviates virus‐induced oxidative stress by limiting ROS accumulation and reinforcing cellular antioxidant capacity.

Given the well‐established crosstalk between oxidative stress and inflammatory signaling [25], we next examined whether SCC‐mediated redox regulation influences cytokine responses during PRRSV infection. RT‐qPCR analysis showed that PRRSV infection significantly increased the transcription of proinflammatory cytokines TNF‐α, IL‐6, and IL‐8 at 12–48 hpi compared with uninfected controls. In contrast, SCC treatment markedly attenuated these responses, such that TNF‐α expression was suppressed from 12 hpi onward (Figure 4H), IL‐8 levels were reduced across all examined time points (Figure 4I), and IL‐6 exhibited a transient elevation at 24 hpi, followed by marked downregulation at later stages (Figure 4J). These results suggest that SCC effectively inhibits PRRSV‐induced inflammatory signaling, likely through the suppression of oxidative stress‐dependent pathways.

### 3.5. Network Pharmacology Analysis Identifies Host Pathways Involved in SCC‐Mediated Antiviral Effects

To further explore the molecular pathways underlying SCC’s antiviral mechanism, a network pharmacology analysis was conducted by integrating PRRSV‐associated host genes with predicted SCC targets. A total of 3369 PRRSV‐related host genes and 115 SCC‐associated targets were collected, and intersection analysis identified 36 overlapping genes potentially involved in SCC‐mediated antiviral effects, including *PPARG*, *IDH1*, *EGFR*, *INS*, and *MAPKs*.

**Figure 5:**
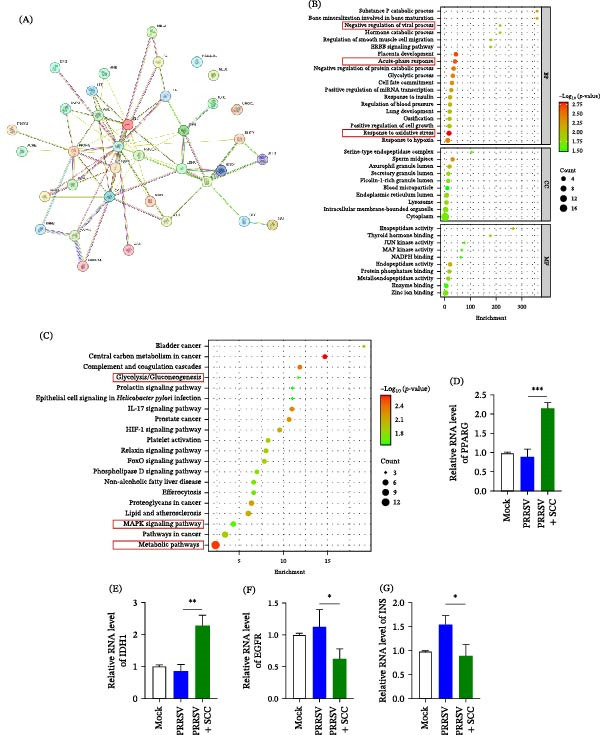
Network pharmacology and RT‐qPCR validation of SCC‐mediated antiviral mechanisms. (A) PPI network of 36 overlapping targets between PRRSV‐related genes and SCC‐associated targets. Node size reflects degree centrality. (B) GO enrichment analysis highlighting major biological processes, including oxidative stress response and regulation of viral processes. (C) KEGG enrichment analysis highlighting major pathways, including glycolysis/gluconeogenesis, MAPK signaling pathway, and metabolic pathways. (D–G) RT‐qPCR validation of representative targets (PPARG, IDH1, EGFR, and INS) in PRRSV‐infected iPAMs with or without SCC treatment. Data are represented as mean ± SD (*n* = 3).  ^∗^, *p* < 0.05;  ^∗∗^, *p* < 0.01;  ^∗∗∗^, *p* < 0.001 versus virus control.

PPI network analysis revealed a network consisting of 36 nodes and 75 edges, with significant enrichment (*p* = 1 × 10^−16^). Degree‐based topological analysis identified *PPARG*, *EGFR*, *INS*, and *IDH1* as central nodes, suggesting that these genes may represent key regulators of SCC’s antiviral activity (Figure 5A). GO enrichment analysis indicated that the overlapping targets were mainly associated with oxidative stress responses, acute‐phase reactions, and negative regulation of viral processes (Figure 5B). KEGG analysis revealed significant enrichment of pathways related to inflammatory regulation and cellular energy metabolism (Figure 5C).

To validate these network‐based predictions, the expression levels of *PPARG*, *IDH1*, *EGFR*, and *INS* were examined in PRRSV‐infected iPAMs following SCC treatment. RT‐qPCR analysis revealed that *PPARG* and *IDH1* were significantly upregulated (Figure 5D–E), suggesting enhanced antioxidant capacity and metabolic regulation. *EGFR* expression was markedly downregulated (Figure 5F), consistent with attenuated inflammatory signaling. Notably, *INS* expression was partially restored toward basal levels (Figure 5G), indicating the recovery of metabolic homeostasis. Collectively, these data suggest that SCC exerts antiviral effects by modulating host pathways related to oxidative stress, inflammation, and metabolic regulation, supporting a multifunctional antiviral mechanism.

### 3.6. SCC Alleviates PRRSV Infection in Piglets

**Figure 6:**
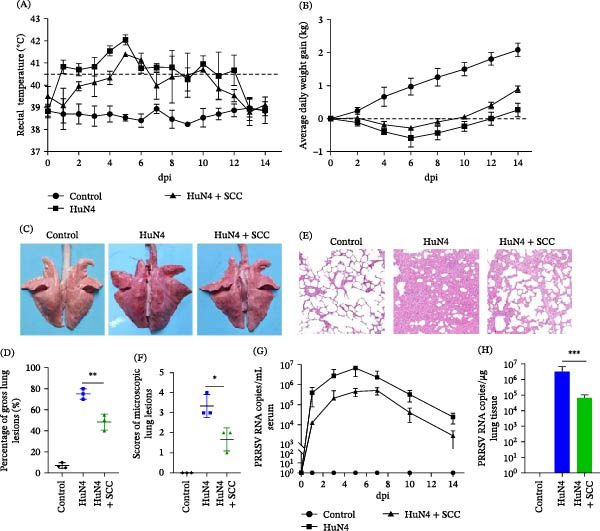
SCC alleviates PRRSV infection in piglets. Piglets were orally administered SCC and subsequently challenged with PRRSV HuN4. (A) Rectal temperature changes were monitored daily following infection. (B) Body weight dynamics of all piglets measured at 0, 2, 4, 6, 8, 10, 12, and 14 dpi. (C) Representative gross lung lesions at 14 dpi. (D) Quantitative gross lung lesion scores. (E) Histopathological analysis of lung tissues by H&E staining at 14 dpi. (F) Mean scores of microscopic lung lesions. (G) PRRSV RNA levels in serum samples measured by RT‐qPCR (one‐tailed *t*‐test). (H) PRRSV RNA levels in lung tissues measured by RT‐qPCR. Data are presented as mean ± SD (*n* = 3).  ^∗^, *p* < 0.05;  ^∗∗^, *p* < 0.01;  ^∗∗∗^, *p* < 0.001 versus virus control.

To assess the in vivo antiviral efficacy of SCC, a controlled PRRSV challenge experiment was conducted in piglets. Rectal temperature monitoring showed that pigs in the HuN4 group developed persistent fever (>40.5°C) starting at 1 dpi and lasting until 12 dpi. In contrast, pigs in the HuN4 + SCC group exhibited a delayed and transient fever response, with body temperatures slightly exceeding 40.5°C only at 5, 6, and 10 dpi. Animals in the uninfected control group remained normothermic throughout the experimental period (Figure 6A). Clinical observations indicated that PRRSV‐infected pigs in both the HuN4 and HuN4 + SCC groups displayed typical symptoms, including dyspnea, fever, and reduced feed intake; however, disease severity was markedly attenuated in SCC‐treated animals. Consistently, SCC‐treated pigs showed a modest, nonsignificant increase in body weight recovery compared with untreated infected piglets at 14 dpi (Figure 6B).

Gross pathological examination at 14 dpi revealed severe pulmonary hemorrhage and edema in HuN4‐infected pigs, whereas only mild edema and limited hemorrhagic lesions were observed in two animals from the HuN4 + SCC group. Lungs from control pigs showed a normal morphology (Figure 6C). Consistent with these observations, quantitative gross lesion scoring revealed significantly lower pulmonary injury scores in the HuN4 + SCC group compared with the HuN4 group (Figure 6D). Histopathological analysis further demonstrated pronounced alveolar wall thickening, erythrocyte infiltration, and inflammatory cell accumulation in the HuN4 group, while alveolar architecture remained largely intact in SCC‐treated piglets (Figure 6E), as further supported by reduced microscopic lung lesion scores (Figure 6F). To determine whether the improvement in clinical manifestations and pulmonary pathology was associated with reduced viral replication, PRRSV RNA levels were quantified by RT‐qPCR. Viral loads in both serum and lung tissues were significantly lower in SCC‐treated piglets than in HuN4‐infected animals (*r* = 0.806, *p* = 0.0143), whereas no viral RNA was detected in the control group (Figure 6G,H). Collectively, these results indicate that oral administration of SCC effectively inhibits viral replication, alleviates clinical symptoms, and mitigates PRRSV‐induced lung pathology in pigs, supporting its potential as a therapeutic candidate for PRRSV control.

## 4. Discussion

PRRSV continues to impose serious economic losses on the swine industry due to its rapid mutation rate, immune evasion, and incomplete protection from current vaccines [7, 26]. Although vaccination remains the cornerstone of PRRSV control, incomplete cross‐protection and limited efficacy against heterologous strains highlight the urgent need for complementary antiviral interventions. In this study, we demonstrate that SCC exerts potent antiviral activity against PRRSV both in vitro and in vivo through a combination of direct virucidal effects and host‐targeted regulatory mechanisms. Importantly, SCC not only suppresses viral replication but also alleviates virus‐induced oxidative stress and inflammatory pathology, addressing the key pathogenic processes of PRRSV disease. These findings position SCC as a promising multifunctional antiviral candidate with translational potential for PRRSV control.

A major finding of this study is that SCC predominantly interferes with the early stages of PRRSV infection. Among the three treatment strategies examined, cotreatment consistently produced the strongest antiviral effect, suggesting that SCC acts during or before viral entry. Mechanistic analyses further demonstrated that SCC inhibits viral adsorption and internalization and directly compromises viral particle integrity. Targeting these early events is particularly important in PRRSV infection, as efficient entry into macrophages is essential for viral immune modulation, establishment of persistence, and subsequent systemic dissemination. By restricting infection initiation, SCC effectively reduces the formation of productive viral infection and the subsequent pathological cascades associated with PRRSV disease.

Notably, the early‐stage antiviral activity observed here is consistent with previous studies showing that SCC and related chlorophyllin derivatives block viral entry, including EV‐A71, coxsackievirus A16 (CV‐A16), influenza A virus (IAV), and human immunodeficiency virus (HIV) [16, 27, 28]. In addition, chlorophyll derivatives have been explored as antiviral interventions in dengue control strategies through vector‐targeted approaches [28]. More recently, comprehensive reviews have highlighted the therapeutic potential of zinc and copper chlorophyllins against respiratory viral infections, including SARS‐CoV‐2, with some formulations advancing into clinical evaluation [15, 18]. Together, these findings support the concept that chlorophyllin‐based compounds possess conserved antiviral properties, particularly against the early stages of viral infection. SCC thus functions as a broad‐spectrum antiviral agent, capable of both extracellular virion inactivation and inhibition of virus‐host attachment, enabling potent suppression of diverse PRRSV strains, including highly pathogenic variants.

Beyond host‐directed effects, SCC exhibited pronounced direct virucidal activity against PRRSV. Incubation of viral particles with SCC resulted in a substantial viral RNA release accompanied by a dramatic loss of infectivity, indicating disruption of viral envelope integrity. Similar virion‐inactivating properties of chlorophyllin derivatives have been reported for poliovirus and bovine herpesvirus replication in vitro [29]. The present study extends these observations to an economically important animal pathogen and suggests that SCC may act through metal‐ion‐mediated destabilization of viral membranes. This dual extracellular and intracellular antiviral action provides a mechanistic explanation for the broad and robust antiviral efficacy observed across multiple PRRSV strains.

PRRSV entry critically depends on the scavenger receptor CD163, which is indispensable for viral uncoating and membrane fusion in macrophages [30, 31]. Notably, SCC significantly reduced CD163 protein levels in both immortalized and primary PAMs without altering transcript levels, suggesting post‐transcriptional regulation. Reduced CD163 expression likely accounts for the observed inhibition of viral entry. Given that genetic deletion or functional inhibition of CD163 confers complete resistance to PRRSV infection [32–35], SCC‐mediated downregulation of this receptor represents a highly effective host‐targeted antiviral strategy. Unlike receptor knockout approaches, pharmacological modulation of CD163 offers a reversible and potentially safer alternative, highlighting the translational significance of this finding.

Accumulating evidence indicates that PRRSV actively induces oxidative stress and inflammatory dysregulation to facilitate viral replication and tissue injury [36]. In this study, SCC significantly reduced intracellular ROS accumulation while simultaneously enhancing the expression of key antioxidant enzymes, including HO‐1, NQO1, and GCLM. Consistent with these observations, previous studies have also reported that SCC can upregulate these antioxidant enzymes [37–40]. Restoration of redox homeostasis is particularly important because oxidative stress not only damages host tissues but also amplifies inflammatory signaling and supports viral replication [41, 42]. By reinforcing antioxidant defenses, SCC creates a cellular environment less permissive to PRRSV propagation. Given the tight link between oxidative stress and inflammation [43], PRRSV infection triggered a robust proinflammatory response. Consistent with its antioxidant effects, SCC markedly attenuated PRRSV‐induced cytokine expression, including TNF‐α, IL‐6, and IL‐8. Excessive cytokine production is a hallmark of PRRSV pathogenesis and contributes directly to lung injury, fever, and clinical disease severity [44, 45]. The ability of SCC to suppress these responses suggests that its antiviral benefit extends beyond viral inhibition to modulate host immunopathology. This dual antiviral and anti‐inflammatory activity is particularly advantageous in PRRSV infection, where disease severity is often driven more by dysregulated host responses than viral load alone.

Network pharmacology analysis further highlighted the multifunctional nature of the SCC. Several key host targets were identified, including PPARG, IDH1, EGFR, and INS, all of which are closely linked to oxidative stress regulation, metabolic homeostasis, and inflammatory signaling. Experimental validation confirmed that SCC upregulated PPARG and IDH1 while suppressing EGFR expression, collectively favoring antioxidant capacity, metabolic balance, and controlled inflammatory signaling. These results suggest that SCC does not act through a single antiviral pathway but instead reshapes the host cellular environment via coordinated regulation of interconnected signaling networks, thereby limiting PRRSV replication and pathogenesis. Notably, these findings are consistent with prior reports showing that PPARG activation plays a key role in regulating antiviral immunity and cytokine production during viral infection [46] and that early modulation of MAPK‐related signaling pathways can effectively suppress PRRSV replication [47].

PRRSV infection is known to reprogram the host cellular metabolism [48, 49]. INS, a key regulator of glucose metabolism, may be exploited by the virus to support its replication, while IDH1 is essential for cellular energy metabolism and redox balance, processes frequently altered during viral infection [50]. Consequently, changes in IDH1 expression or activity may contribute to host antioxidant responses and influence viral replication or pathogenicity [51]. However, it should be noted that network pharmacology predictions are inherently based on existing databases and in silico algorithms, which may not fully capture the complexity and context‐specific dynamics of host‐virus interactions. Therefore, the identified targets and pathways should be interpreted with caution, as false‐positive associations cannot be excluded and current evidence remains largely correlative. In addition, given the multitarget nature of SCC, it is challenging to definitively determine the specific contribution of individual targets (e.g., INS and IDH1) using single‐target approaches such as inhibitors or siRNA‐mediated knockdown as compensatory effects across interconnected pathways may occur. Future studies combining targeted perturbation strategies (e.g., siRNA or pharmacological modulators) with systems‐level approaches, such as CRISPR screening or multiomics integration, will be necessary to define the causal contributions of individual targets within the SCC‐mediated antiviral network.

Importantly, the antiviral effects of SCC observed in vitro were confirmed in a PRRSV challenge model in piglets. Oral administration of SCC significantly reduced viral loads in serum and lung tissues, alleviated fever, promoted body‐weight recovery, and markedly attenuated pulmonary pathological lesions. Quantitative histopathology and network analyses suggest that these protective effects may involve modulation of inflammatory responses and host metabolic pathways, although the precise mechanisms remain to be elucidated. No adverse reactions were observed during the experiment. However, only a single SCC dose was evaluated in vivo, limiting the ability to define the therapeutic window. Future studies should include dose–response analyses and optimized regimens to determine the minimal effective dose and establish safety margins, while also addressing cost‐effectiveness, formulation, and regulatory feasibility. In addition, SCC may have potential as an adjunct to existing PRRSV vaccines, and combination strategies warrant further evaluation under field conditions. These in vivo findings are particularly notable, as many antiviral compounds that are effective in cell culture fail to confer protection in animals, highlighting the feasibility of SCC as an orally administered antiviral agent for practical applications.

In conclusion, this study demonstrates that SCC is a multifunctional antiviral agent capable of suppressing PRRSV infection through both virion‐directed and host‐directed mechanisms. By simultaneously limiting viral entry, disrupting viral integrity, and mitigating oxidative and inflammatory pathology, these mechanisms collectively explain the observed in vivo outcomes, including reduced viral loads, alleviated clinical symptoms, and attenuated lung lesions. These findings not only provide mechanistic insight into the antiviral activity of chlorophyllin derivatives but also identify SCC as a promising therapeutic candidate for PRRSV control.

## Author Contributions

**Xinrong Wang:** data curation, formal analysis, investigation, methodology, writing – original draft. **Juan Zhang:** data curation, investigation, resources, software. **Li Rui, Meiyu Jia, and Lizhi Fu:** data curation, methodology. **Junhai Zhu:** formal analysis, methodology. **Nan Yan:** resources, software. **Longxiang Zhang:** data curation, formal analysis. **Yue Wang:** formal analysis, investigation, methodology, writing – review and editing.

## Funding

This study was supported by grants from the National Natural Science Foundation of China (Grants 32573404 and 32302852), the Natural Science Foundation of Chongqing (Grant CSTB2024NSCQ‐MSX0467), the Fundamental Research Funds for the Central Universities (Grant SWU‐KR22036), and the Doctoral Research Innovation Program of Chongqing (Grant CYB240137).

## Ethics Statement

All animal procedures were approved by the Institutional Animal Care and Use Committee (IACUC) of Southwest University (Approval Number LAC2025‐2‐0259) and conducted in strict accordance with the Guidelines for the Care and Use of Laboratory Animals issued by Southwest University.

## Conflicts of Interest

The authors declare no conflicts of interest.

## Data Availability

Data sharing is not applicable to this article as no datasets were generated or analyzed during the current study.
